# Novel Mutation of Tyrosinemia in a Child With Hypophosphatemic Rickets

**DOI:** 10.1097/PG9.0000000000000176

**Published:** 2022-02-25

**Authors:** Surabhi Dogra, Karunesh Kumar, Rohan Malik, Smita Malhotra, Anupam Sibal

**Affiliations:** From the * Department of Pediatric Gastroenterology, Indraprastha Apollo Hospital, New Delhi, India; † Royal Children’s Hospital, Melbourne, VIC, Australia; and; ‡ Division of Pediatric Gastroenterology, Department of Pediatrics, All India Institute of Medical Sciences, New Delhi, India.

**Keywords:** renal tubulopathy, hypotonia, deranged coagulation, liver transplant

## Abstract

Tyrosinemia is an inherited metabolic disease of fumarylacetoacetate enzyme. A male infant presented to us with clinical features of rickets, floppiness, and a deranged coagulation profile. A novel mutation causing Tyrosinemia was discovered on the basis of genetic sequencing.

## INTRODUCTION

Tyrosinemia type I usually presents as acute liver failure or liver dysfunction with renal tubular defects, growth failure, and rickets. High urine succinyl acetone levels and genetic sequencing aid in the diagnosis. NTBC [2-(2-nitro-4-trifluoromethylbenzoyl)-1,3-cyclohexanedione] or Nitisinone, the only effective medical therapy, is expensive and not readily available in India. Initiation of therapy should be soon after birth to prevent the development of hepatocellular carcinoma. Nitisinone and low-tyrosine diet result in >90% survival rate, normal growth, improved liver function, prevention of cirrhosis, correction of renal tubular acidosis, and improvement in secondary rickets (1). We report a child with refractory rickets diagnosed to have tyrosinemia due to a novel mutation on genetic sequencing.

## CASE

Our patient was born at term with a birth weight of 2.5 kg to nonconsanguineous parents. The neonatal period was uneventful. Baby did not develop any cholestasis or coagulopathy and newborn screening for genetic disorders was not done. The infant was hospitalized for a lower respiratory tract infection at 5 months of age, when features of rickets and hepatomegaly were observed.

At 9 months, he presented with progressive widening of wrists, prominent ribs, and abdominal distention. Mother complained of inadequate weight gain with delayed age-appropriate milestones as compared to his elder sibling. She noticed floppiness and increased frequency of urination.

Investigations revealed normal counts and rest as below (Table 1). Radiographs of knee and wrist were suggestive of rickets. Fractional excretion of phosphate (Fe-PO_4_) and tubular reabsorption of phosphate (TrP) was [“were”] suggestive of proximal tubulopathy and urine analysis detected glycosuria. Normal anion gap metabolic acidosis was treated with oral bicarbonate supplements.

**TABLE 1.** Investigations

	Pretransplant (9 months of age)	3 months posttransplant
Serum calcium	9 mg/dL	9.1 mg/dL
Serum phosphorus	1.3 mg/dL	3 mg/dL
Alkaline phosphate	2669 mg/dL	760 mg/dL
AST	56 IU/mL	28 IU/mL
ALT	27 IU/mL	32 IU/mL
Albumin	4.1 g/dL	4 g/dL
Total bilirubin	1.31 mg/dL	0.6 mg/dL
INR	1.9	1.5
Vitamin D	9.3 ng/dL	134.41 ng/dL
Serum AFP	1,97,914 ng/mL	23 ng/mL
Serum creatinine	0.1 mg/dL	0.3 mg/dL
Urinary creatinine	20.3 mg/dL	11.48 mg/dL
Urinary phosphate	110.48 mg/dL	6 mg/dL
Anthropometry		
Weight	6.5 kg	9 kg
Height	63 cm	65 cm

AFP = alpha fetoprotein; INR = International normalized ratio.

**FIGURE 1.**
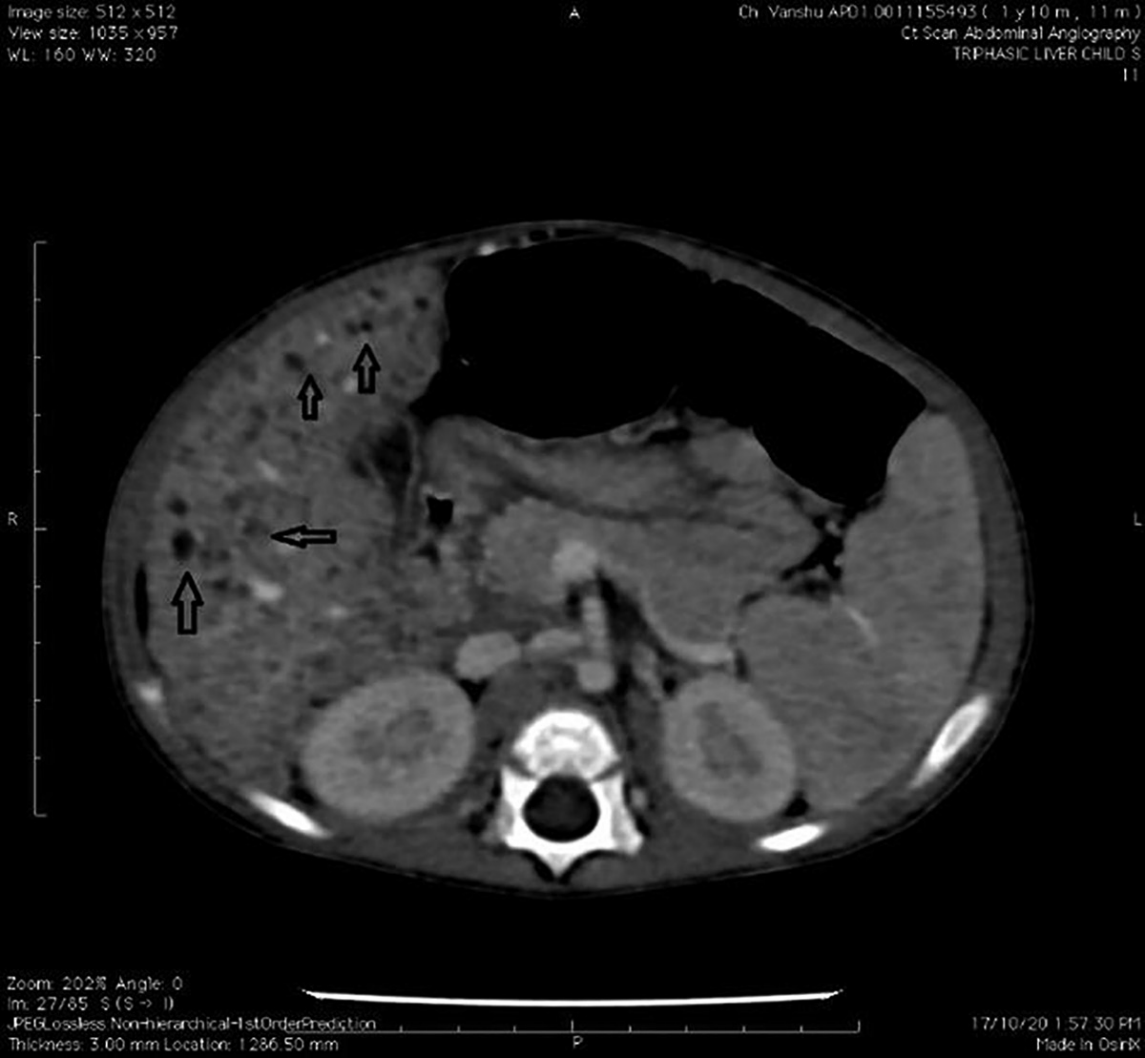
Contrast enhanced computerized tomography axial images through the abdomen revealed heterogenous liver attenuation with multiple non-enhancing hypodense nodules in both the lobes of liver.

Ultrasonography revealed heterogenous echotexture of the liver and multiple hypoechoic nodules, largest being 1.7 cm in segment V of the liver. Triple phase CT revealed multiple small hypodense nodules of varying size present in both lobes of the liver in venous phase and delayed phase (Fig. 1).

Tandem mass spectrometry and gas chromatography and mass spectrometry screening of urine and blood revealed raised level of succinylacetone (7.3 µM/L, normal is <2), suggestive of tyrosinemia. It was further confirmed by spectophotometric analysis of urine, which showed level of 480 µM/L (normal <20 µM/L). Limited exome sequencing of common pathogenic genes relevant to the clinical condition was sent. It revealed a homozygous 3′ splice site variation in intron 4 of the **FAH gene** (chr15:g.80160408A>G) that affects the invariant AG acceptor splice site of exon 4 (c.315-2A>G; ENST00000407106.5).

Living donor liver transplant (father being the donor) was done at 1 year of age as the presence of multiple large nodules with high serum AFP levels raised concerns about malignancy, also the family expressed inability to maintain on NTBC due to availability and cost issues. The explant liver revealed micro and macronodular cirrhosis, with few discrete nodules, most likely regenerative nodules, which were cellular and composed of monotonous cell population mostly hepatocytes. There was no evidence of malignancy. The child is well 6 months after transplant, AFP levels are within normal range on regular follow up.

## DISCUSSION

Hepatorenal tyrosinemia type 1 is an inborn error of metabolism affecting the liver, kidneys, and the peripheral nerves. It manifests in infancy as acute liver failure, refractory rickets, bleeding diathesis, renal tubular acidosis, or neurologic crisis, and there is failure to thrive. Cirrhosis of liver, hepatocellular carcinoma, and death usually occur in the early course of life. The most distinguishing characteristic of type I tyrosinemia is liver and kidney involvement (2), as was observed in our patient.

Children present with coagulation abnormalities, PT and aPTT, are markedly prolonged but not correctable by Vit K supplementation. Transaminases and bilirubin are usually only marginally elevated, serum albumin levels fall when synthetic dysfunction sets in with disease progression. Our patient had marginally raised transaminases and bilirubin, deranged INR with normal serum albumin levels.

The child presented with frontal bossing, widening of wrists, beading of ribs, knock knees, and delayed dentition, diagnosed as refractory rickets. Hypophosphatemic rickets due to proximal tubulopathy was diagnosed because of increased phosphate losses in urine, glucosuria, and normal anion gap metabolic acidosis on blood gas analysis.

Rickets being a common presentation of tyrosinemia, a sizeable number of children present with it due to proximal tubulopathy like our patient. Nuclear medicine (DTPA) renal scan done before the transplant showed normal glomerular filtration rate. In a study conducted on 32 tyrosinemia type I patients, nephromegaly (47%), hyper echogenicity of kidneys (47%), and nephrocalcinosis (16%), aminoaciduria (82%), hypercalciuria (67%), tubular acidosis (59%), decreased glomerular filtration rate (48%) were the common features found (3).

Urinary succinylacetone level measurement is a sensitive and specific test for the diagnosis of tyrosinemia. FAH gene on chromosome 15 is affected in tyrosinemia, and gene sequencing is recommended for confirmation. About 278 variants in FAH gene have been identified, of which 93 variants are pathogenic or likely pathogenic. Our patient had homozygous mutation, classified as likely pathogenic. This variant was a novel mutation and has not been reported in the 1000 genomes, gnomAD, and laboratory’s internal databases. Mutation occurred in Intron 4 of FAH gene that affects the invariant AG acceptor splice site of exon 4, never previously reported. The most common mutation is IVS12 + 5(G→A), which is a mutation in the splice site consensus sequence of intron 12, therefore affecting exon 12. A second allele is the IVS6-1(G-T) mutation. This mutation results in a nonfunctional enzyme (4). Since the first report of the missense mutation n161 in the FAH mRNA, many mutations have been identified causing the disease (5,6). Of these, there are 4 common mutations observed in subjects from the Indian subcontinent (5).

Children diagnosed early show good outcome on NTBC. Initiation of therapy in later stages of the disease, especially if dysplasia and malignant transformation have set in, leads to failure of medical management. Moreover, poor availability and prohibitive cost preclude NTBC use in many countries like ours, necessitating liver transplantation.
